# Novel Strategies to Enhance Vaccine Immunity against Coccidioidomycosis

**DOI:** 10.1371/journal.ppat.1003768

**Published:** 2013-12-19

**Authors:** Garry T. Cole, Chiung-Yu Hung, Sam D. Sanderson, Brady J. Hurtgen, Marcel Wüthrich, Bruce S. Klein, George S. Deepe, Gary R. Ostroff, Stuart M. Levitz

**Affiliations:** 1 Department of Biology and South Texas Center for Emerging Infectious Diseases, University of Texas at San Antonio, San Antonio, Texas, United States of America; 2 Department of Pharmaceutical Sciences, University of Nebraska Medical Center, Omaha, Nebraska, United States of America; 3 U.S. Army Institute of Surgical Research, Fort Sam Houston, San Antonio, Texas, United States of America; 4 Department of Pediatrics and Microbiology and Immunology, University of Wisconsin, School of Medicine and Public Health, Madison, Wisconsin, United States of America; 5 Division of Infectious Diseases, College of Medicine, University of Cincinnati and Veterans Affairs Hospital, Cincinnati, Ohio, United States of America; 6 Department of Medicine, University of Massachusetts Medical School, Worcester, Massachusetts, United States of America; Duke University Medical Center, United States of America

## A Primary Pathogen of Mammalian Hosts

Coccidioidomycosis is a potentially life-threatening respiratory mycosis endemic to the Americas and caused by inhalation of spores produced by the molds *Coccidioides immitis* and *C. posadasii*. The dry, air-dispersed infectious spores (arthroconidia) are derived from saprobic-phase filaments that grow in semidesert soil of the southwestern United States and arid regions of Mexico and Central and South America [1]. *Coccidioides* is a dimorphic ascomycetous fungus with distinct saprobic and parasitic phases and is classified in the order Onygenales together with other genera of pathogenic molds that include *Histoplasma*, *Blastomyces*, and *Paracoccidioides*. The clinical spectrum of disease caused by these environmental pathogens ranges from a mild infection that resolves spontaneously to a disseminated mycosis. Inhaled spores of *Coccidioides* are small enough to colonize the lowermost reaches of the respiratory tree. Nonhuman primates experimentally challenged by aerosolization with fewer than ten infectious propagules developed a severe form of the disease and died within four to six weeks [2]. The parasitic cycle of *Coccidioides* is unique among these medically important, dimorphic molds. Spores in the lungs germinate to form multinucleate spherules (>60 µm diameter) that are too large to be engulfed by host phagocytes. The contents of mature spherules differentiate into a multitude of propagative endospores. Production of indigestible spherules that display high fecundity during the endosporulation stage confound phagocytic defenses and augment survival of the fungus in the lungs. Mammalian innate immune defenses are further compromised by the ability of endosporulating spherules to generate an alkaline microenvironment at infection sites due to their release of ammonia, which contributes to the pathogen's virulence [3].

## Need for a Vaccine

The incidence of symptomatic coccidioidomycosis in the United States increased from 2,265 reported cases in 1998 to 22,401 in 2011 based on data from the National Notifiable Diseases Surveillance System (NNDSS) [4]. This database likely underestimates the actual burden of disease. Reporting cases of Valley Fever is not mandated in every state within known areas of endemicity. Moreover, *Coccidioides* has established niches in nontraditional endemic regions not considered in the report [5]. Approximately 10% of the total United States population resides in the Southwest, including more than 300 thousand military personnel who train in the semidesert terrain. Racial heritage (e.g., African and Filipino) influences susceptibility to this mycosis, although the immunologic mechanism is unclear [6]. Millions of tourists also visit popular landmarks in Texas, Arizona, and southern California, and although many people are exposed to *Coccidioides* spores, the majority (>60%) do not develop symptomatic disease. Nevertheless, a significant number of these individuals are diagnosed with reactivated infection months to years after returning home. Risk of infection is particularly high during dust storms, which occur in the spring and summer in endemic regions (http://directorsblog.health.azdhs.gov/?tag=valley-fever). A vaccine against coccidioidomycosis would arguably promote the well-being of at-risk populations in the United States in addition to people who reside in arid areas of Latin America. A compelling argument for the feasibility of such a vaccine is based on retrospective clinical evidence that individuals who recover from symptomatic coccidioidal infection remain skin-test positive and likely acquire life-long immunity to the disease. Both clinical data and results of experimental animal studies have shown that T-cell immunity is essential for protection against coccidioidomycosis and mammalian hosts with deficiency of CD4^+^ T cells are at elevated risk of contracting the respiratory disease [7]. A vaccine development strategy must, therefore, be designed to protect immunocompetent and as well as immunocompromised individuals. A formalin-killed spherule vaccine was tested in a clinical trial between 1980 and 1985 and, although there was a trend toward reduced disease, reactogenicity at sites of immunization was too great, requiring the vaccine dose to be reduced and thus underpowered [8]. Nevertheless, this early pioneering study laid the foundation for current efforts to generate a human vaccine.

## A Novel Adjuvant Enhances Cellular Immunity Induced by a Live Vaccine

For many diseases in which natural infection confers protective immunity against reinfection, live vaccines have been successful in promoting robust and durable immune defenses in humans. An attenuated mutant of *Coccidioides* was generated by disruption of two chitinase genes that resulted in the inability of the organism to endosporulate [9]. Inbred mice immunized subcutaneously with the attenuated strain all survived after intranasal challenge with a potentially lethal spore inoculum. However, sterile immunity was not achieved and pulmonary tissue damage associated with a persistent host inflammatory response was evident, especially in BALB/c mice. Neutrophil migration to infected lungs plays a pivotal role during the early protective response [10], but persistence of these innate immune cells in the presence of elevated inflammatory cytokines IL-1β, IL-1α, and TNF-α leads to acute lung injury and disease exacerbation. BALB/c and C57BL/6 mouse strains differ in their ability to regulate inflammation associated with coccidioidal lung infection, possibly simulating a clinical difference that partly accounts for the heterogeneity of human immune responses to coccidioidomycosis. Infected BALB/c mice that had been immunized with the live, attenuated vaccine consistently revealed 8-fold higher numbers of lung-infiltrated neutrophils and significantly increased fungal burden compared to vaccinated C57BL/6 mice [9], [11], [12]. Thus, excessive inflammation due to persistent high neutrophil counts appears to compromise vaccine immunity.

**Figure 1:**
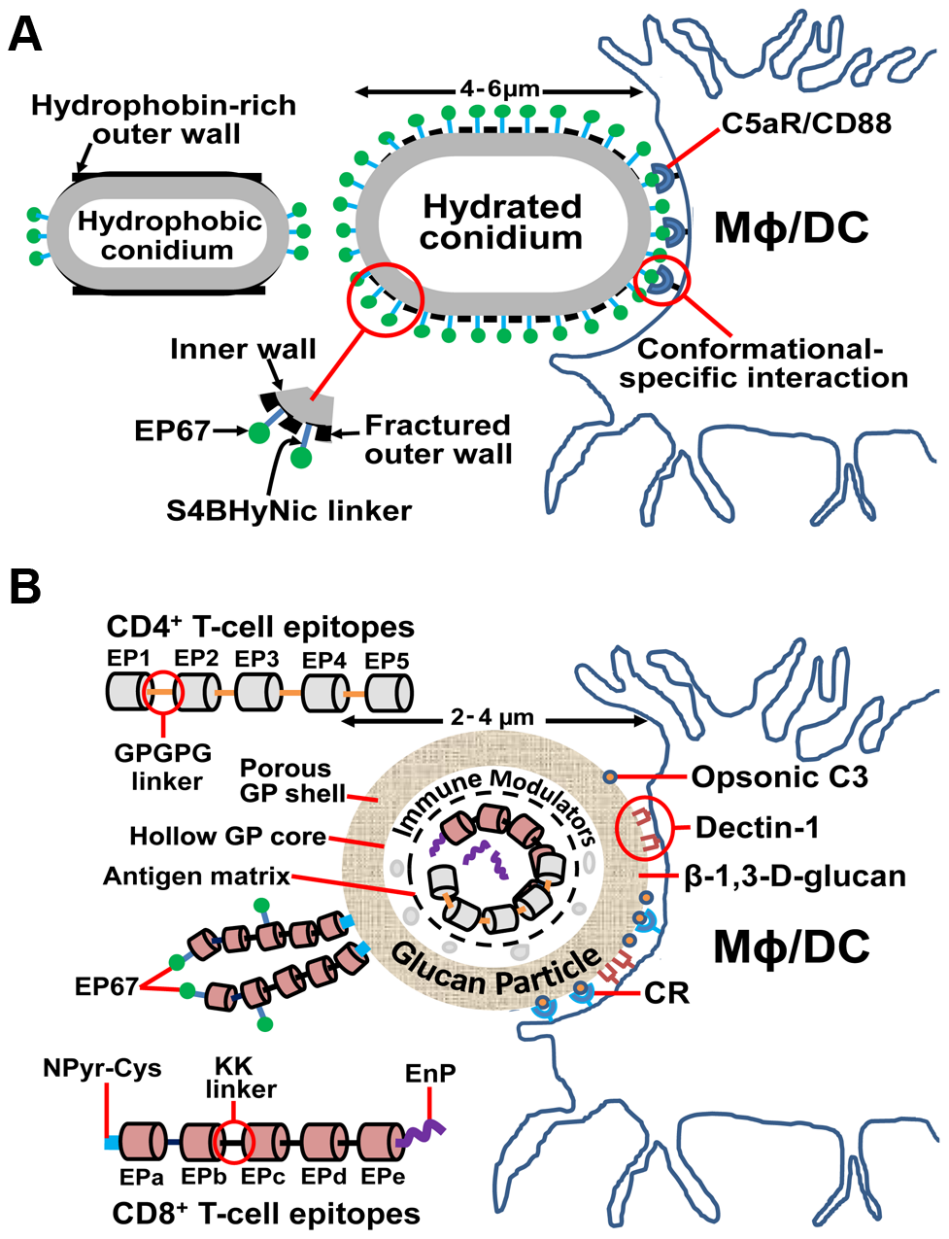
Novel adjuvant and vaccine delivery system for enhancement of protective immunity to coccidioidomycosis. (A) An agonist of human complement fragment C5a (EP67) bound to live cells of an attenuated vaccine strain of *Coccidioides* enhances T-cell immunity while downregulating inflammatory pathology. Hydration and dilation of live arthroconidia of the vaccine strain results in fracture of the hydrophobic outer conidial wall and exposure of lysine residues of the underlying inner wall that can conjugate with EP67 via S4BHyNic linkers (succinimidyl-4-benzoylhydrazino-nicotinamide). The concept is that the engineered conformational features of EP67 direct the conjugated vaccine to C5aR/CD88 receptors on DC and MΦ, but not neutrophils, and activate phagocytosis, antigen processing, and presentation by APCs while dampening persistent neutrophil-associated inflammation at infection sites [12], [13]. **(B) A glucan particle (GP)-based subunit vaccine platform combines adjuvanicity with antigen delivery to induce robust and durable CD4^+^ and CD8^+^ T-cell responses to** ***Coccidioides*** **infection.** GPs are composed of a porous shell and hollow core. The protein vaccine is co-loaded with a carrier protein and interacted with yeast RNA within the core to form an antigen complex that is too large to diffuse out through the shell. Endo-Porter (EnP) peptides added to the antigen matrix enhance release of the CD8^+^ vaccine proteins to the cytoplasm of APCs after phagocytosis of GPs and promote processing and presentation of MHC I complexes, resulting in activation of protective CD8^+^ T cells. Between the antigen matrix and shell is a GP layer that can accommodate immune modulators (e.g., CpG, siRNA) that help to elicit a protective response to infection. Multiepitope constructs also may be coupled to the surface of GPs by addition of a pyridyl-S-cysteine peptide at the N-terminus (NPyr-Cys) of the vaccine protein. The C-terminus of GP surface-coupled vaccine proteins may be conjugated with EP67 as in Figure 1A. EP67 directs the EBV to complement receptors and, although not yet tested, may further enhance Th17 immunity. CD4^+^ and CD8^+^ multiepitope vaccines are constructed using nonimmunogenic GPGPG or di-lysine (KK) linkers [25], [26]. β-1,3-glucans and opsonic C3 deposited on the surface of GPs bind to Dectin-1 and complement receptors (CR), respectively, and induce phagocytosis by APCs (MΦ and DC) followed by antigen processing and peptide epitope display as MHC I or II complexes [17], [19].

We speculated that if the protracted recruitment of neutrophils to infection sites could be downregulated in BALB/c mice, the outcome could be improved. We accomplished this using a novel adjuvant conjugated with the live *Coccidioides* vaccine. A decapeptide agonist of the biologically active C-terminal region of human complement component C5a functions as an adjuvant that enhances antigen processing by antigen presenting cells (APCs) and promotes cellular immunity. Unique conformational features of this agonist allow it to bind with high affinity to C5a receptors (C5aR/CD88) on macrophages (MΦ) and dendritic cells (DC) but not neutrophils [13]. The adjuvant, termed EP67, was conjugated with lysine residues on the surface of live arthroconidia from the vaccine strain [14] (Figure 1A). Spore hydration and dilation steps physically fractured the cysteine-rich, hydrophobic outer wall, permitting EP67 conjugation with underlying cell wall components. EP67 directs the vaccine to C5aR-bearing MΦ and DC, inducing phagocytosis and antigen presentation. BALB/c mice immunized with the EP67-conjugated, live vaccine revealed increased survival, reduction of inflammatory pathology, lower fungal burden, and decreased recruitment of neutrophils to the lungs at 11 days post-challenge compared to mice immunized with the nonconjugated vaccine. The EP67 adjuvant enhanced vaccine protection by augmenting T-cell immunity, especially T-helper 1 (Th1) and Th17 responses [12]. Th17 immunity is indispensable for protection against coccidioidomycosis [11]. Although not yet approved for use in humans, EP67 is a promising adjuvant that drives relevant pathways of protective immunity to *Coccidioides* infection. However, in spite of these positive results, live vaccines are not ideal for immunocompromised individuals at risk of severe reaction to the attenuated strain, which highlights the need for safer and more efficacious vaccines using purified antigens, appropriate adjuvants, and efficient antigen delivery systems.

## An Innovative Adjuvant and Delivery Platform Contributes to the Protective Efficacy of Alternative Subunit Vaccines

Although CD4^+^ T cells are central to the mammalian protective response to *Coccidioides* infection, CD8^+^ T cells also mediate vaccine immunity [15]. Recent studies of infections caused by other dimorphic fungal pathogens have revealed that even in the absence of CD4^+^ T-cell help vaccine-induced IL-17–producing CD8^+^ T cells (Tc17) show long-term survival and confer protection [16]. This plasticity of cellular immunity argues for the feasibility of developing a vaccine against coccidioidomycosis in patients with immune deficiencies, including those with few or no CD4^+^ T cells. Our strategy in the design of a subunit vaccine has been to generate polypeptide constructs containing multiple immunodominant CD4^+^ or CD8^+^ T-cell epitopes that bind promiscuously to human major histocompatibility complex class II (MHC II) and MHC I molecules, respectively. T-cell epitopes were initially predicted by immunoinformatic sequence analysis of a selection of the many antigenic proteins of *Coccidioides* shown to induce protection against coccidioidal lung infections in mice [17]. Immunoreactivity of synthetic epitope peptides was validated by human MHC-specific binding assays and stimulation of immune T-cells *in vitro* to produce protective Th1 and Th17 cytokines. Epitope-based vaccines (EBV) against microbial diseases have been shown to be immunologically potent, relatively easy to produce, and are predicted to be safe in humans [18]. Bacterial-expressed recombinant CD4^+^ and CD8^+^ T-cell epitopes joined by nonimmunogenic linkers have been loaded into glucan particles (GPs), which deliver the EBV to APCs and serve as a potent adjuvant system [19] (Figure 1B). The selected epitopes are conserved between *C. immitis* and *C. posadasii* [17]. GPs are purified, hollow, porous shells made by alkali and acid extraction of baker's yeast, composed primarily of β-1,3-glucans, and essentially devoid of proteins, lipids, and mannans. GPs activate the alternative pathway of complement, leading to deposition of C3 fragments. GPs are phagocytosed by MΦ and DC via complement receptors and Dectin-1. Epitope-based vaccines composed of CD4^+^ or CD8^+^ epitopes can either be loaded into GPs or conjugated to the GP surface. EP67 conjugation with epitope-based protein vaccines may provide an effective mechanism to further augment Th17 immunity. CD8^+^ epitope–based vaccines internalized by DC and presented as peptide-MHC class I complexes can be enhanced by binding the EBV to a weak-base amphiphilic peptide (Endo-Porter) that promotes antigen release into the cytoplasm of APCs [20]. The GP core can accommodate various adjuvant classes to fine-tune immune responses and generate optimal and durable protection. The core of the loaded GP forms a central antigen matrix with a surrounding layer that accommodates immune modulators. For example, an oligodeoxynucleotide adjuvant (CpG) loaded into a GP-internalized, CD4^+^ epitope preparation and used to immunize mice against intranasal infection significantly improved protection compared to the same vaccine admixed with CpG in the absence of GPs [17]. Further, Endo-Porter has been shown to form stable nanocomplexes with small interfering RNAs (siRNAs), which when loaded into GPs and delivered to phagocytic cells can downregulate proinflammatory signaling and curb inflammatory pathology [21]. The glucan particle delivery and adjuvant platform offers multiple options for application of molecular tools that can optimally tailor a human epitope-based vaccine against coccidioidomycosis. Corporate partners currently evaluating the GP delivery technology are planning to carry out prerequisite toxicology and safety studies prior to the submission of an application for human testing.

## Future Directions

Development of an effective coccidioidomycosis vaccine faces several major challenges. Reported studies to date have assumed that eliciting cell-mediated immunity will translate to protection against the disease. However, the role of humoral response to infection is still largely unexplored. It may be possible to protect inbred mice with a vaccine that elicits cell-mediated immunity alone, but we cannot assume that this observation will translate to humans. A recent vector-based vaccine against tuberculosis intended to protect by eliciting strong CMI failed in humans despite showing efficacy in mice [22]. Most cases of coccidioidomycosis recorded in the United States occur in individuals over 60 years of age [4]. Vaccine development for the elderly must take into account the aging immune system, a condition that may increase vulnerability to infectious diseases [23]. A coccidioidomycosis vaccine potentiated by an adjuvant specifically designed to stimulate the immune system of this demographic group (e.g., EP67) [24] is a current research priority.

## References

[1] Fisher FS, Bultman MW, Johnson SM, Pappagianis D, Zaborsky E (2007) *Coccidioides* niches and habitat parameters in the southwestern United States: a matter of scale. Ann N Y Acad Sci 1111: 47–72. 17344527 10.1196/annals.1406.031

[2] Blundell GP, Castleberry MW, Lowe EP, Converse JL (1961) The pathology of *Coccidioides immitis* in the *Macaca mulatta* . Am J Pathol 39: 613–630. 13870259 PMC1942437

[3] Wise HZ, Hung CY, Whiston E, Taylor JW, Cole GT (2013) Extracellular ammonia at sites of pulmonary infection with *Coccidioides posadasii* contributes to severity of the respiratory disease. Microb Pathog 59–60: 19–28. 10.1016/j.micpath.2013.04.003 PMC3656146 23583291

[4] Anonymous (2013) Increase in reported coccidioidomycosis - United States, 1998–2011. Morb Mortal Wkly Rep 62: 217–221. PMC4605002 23535687

[5] Marsden-Haug N, Goldoft M, Ralston C, Limaye AP, Chua J, et al (2013) Coccidioidomycosis acquired in Washington State. Clin Infect Dis 56: 847–850. 23223598 10.1093/cid/cis1028

[6] Ruddy BE, Mayer AP, KO MG, Labonte HR, Borovansky JA, et al (2011) Coccidioidomycosis in African Americans. Mayo Clin Proc 86: 63–69. 21193657 10.4065/mcp.2010.0423 PMC3012635

[7] Cole GT, Hurtgen BJ, Hung C-Y (2012) Progress toward a human vaccine against coccidioidomycosis. Curr Fungal Infect Rep 6: 235–244. 23585916 10.1007/s12281-012-0105-y PMC3620201

[8] Pappagianis D (1993) Evaluation of the protective efficacy of the killed *Coccidioides immitis* spherule vaccine in humans. The Valley Fever Study Group. Am Rev Respir Dis 148: 656–660. 8368636 10.1164/ajrccm/148.3.656

[9] Xue J, Chen X, Selby D, Hung-Y, Yu J-J, Cole GT (2009) A genetically engineered live attenuated vaccine of *Coccidioides posadasii* protects BALB/c mice against coccidioidomycosis. Infect Immun 77: 3196–3208. 19487479 10.1128/IAI.00459-09 PMC2715678

[10] Balamayooran T, Batra S, Balamayooran G, Cai S, Kobayashi KS, et al (2011) Receptor-interacting protein 2 controls pulmonary host defense to *Escherichia coli* infection via the regulation of interleukin-17A. Infect Immun 79: 4588–4599. 21844230 10.1128/IAI.05641-11 PMC3257916

[11] Hung C-Y, Gonzalez A, Wüthrich M, Klein BS, Cole GT (2011) Vaccine immunity to coccidioidomycosis occurs by early activation of three signal pathways of T helper cell response (Th1, Th2, and Th17). Infect Immun 79: 4511–4522. 21859851 10.1128/IAI.05726-11 PMC3257932

[12] Hung C-Y, Hurtgen BJ, Bellecourt M, Sanderson SD, Morgan EL, et al (2012) An agonist of human complement fragment C5a enhances vaccine immunity against *Coccidioides* infection. Vaccine 30: 4681–4690. 22575167 10.1016/j.vaccine.2012.04.084 PMC3372706

[13] Morgan EL, Morgan BN, Stein EA, Vitrs EL, Thoman ML, et al (2009) Enhancement of *in vivo* and *in vitro* immune functions by a conformationally biased, response-selective agonist of human C5a: implications for a novel adjuvant in vaccine design. Vaccine 28: 463–469. 19836478 10.1016/j.vaccine.2009.10.029 PMC2789185

[14] Phillips JA, Morgan EL, Dong Y, Cole GT, McMahan C, et al (2009) Single-step conjugation of bioactive peptides to proteins via a self-contained succinimidyl bis-arylhydrazone. Bioconjug Chem 20: 1950–1957. 19788175 10.1021/bc9002794 PMC2889006

[15] Fierer J, Waters C, Walls L (2006) Both CD4^+^ and CD8^+^ T cells can mediate vaccine-induced protection against *Coccidioides immitis* infection in mice. J Infect Dis 193: 1323–1331. 16586371 10.1086/502972

[16] Nanjappa SG, Heninger E, Wüthrich M, Gasper DJ, Klein BS (2012) Tc17 cells mediate vaccine immunity against lethal fungal pneumonia in immune deficient hosts lacking CD4^+^ T cells. PLoS Pathog 8: e1002771 doi:10.1371/journal.ppat.1002771 22829762 10.1371/journal.ppat.1002771 PMC3400565

[17] Hurtgen BJ, Hung C-Y, Ostroff GR, Levitz SM, Cole GT (2012) Construction and evaluation of a novel recombinant T cell epitope-based vaccine against coccidioidomycosis. Infect Immun 80: 3960–3974. 22949556 10.1128/IAI.00566-12 PMC3486067

[18] Rosa DS, Ribeiro SP, Cunha-Neto E (2010) CD4^+^ T cell epitope discovery and rational vaccine design. Arch Immunol Ther Exp (Warsz) 58: 121–130. 20155490 10.1007/s00005-010-0067-0

[19] Huang H, Ostroff GR, Lee CK, Specht CA, Levitz SM (2010) Robust stimulation of humoral and cellular immune responses following vaccination with antigen-loaded β-glucan particles. MBio 1: e00164–10. 20802824 10.1128/mBio.00164-10 PMC2925077

[20] Ikeuchi N, Futami J, Hosoi A, Noji S, Kurachi M, et al (2010) Efficient cross-presentation of soluble exogenous antigens introduced into dendritic cells using a weak-based amphiphilic peptide. Biochem Biophys Res Commun 392: 217–222. 20067764 10.1016/j.bbrc.2010.01.019

[21] Tesz GJ, Aouadi M, Prot M, Nicoloro SM, Boutet E, et al (2011) Glucan particles for selective delivery of siRNA to phagocytic cells in mice. Biochem J 436: 351–362. 21418037 10.1042/BJ20110352

[22] Tameris MD, Hatherill M, Landry BS, Scriba TJ, Snowden MA, et al (2013) Safety and efficacy of MVA85A, a new tuberculosis vaccine in infants previously vaccinated with BCG: a randomized, placebo-controlled phase 2b trial. Lancet 381: 1021–1028. 23391465 10.1016/S0140-6736(13)60177-4 PMC5424647

[23] Rappuoli R, Mandl CW, Black S, De Gregorio E (2011) Vaccines for the twenty-first century society. Nat Rev Immunol 11: 865–872. 22051890 10.1038/nri3085 PMC7098427

[24] Morgan EL, Thoman ML, Sanderson SD, Phillips JA (2010) A novel adjuvant for vaccine development in the aged. Vaccine 28: 8275–8279. 20965299 10.1016/j.vaccine.2010.10.008 PMC2997863

[25] Livingston B, Crimi C, Newman M, Higashimoto Y, Appella E, et al (2002) A rational strategy to design multiepitope immunogens based on multiple Th lymphocyte epitopes. J Immunol 168: 5499–5506. 12023344 10.4049/jimmunol.168.11.5499

[26] Yano A, Onozuka A, Asahi-Ozaki Y, Imai S, Hanada N, et al (2005) An ingenious design for peptide vaccines. Vaccine 23: 2322–2326. 15755620 10.1016/j.vaccine.2005.01.031

